# Multiplex Species-Specific PCR Identification of Native Onchidiid Slugs in the South China Sea: A Useful Tool for Application in Onchidiid Slug Stock Management and Germplasm Conservation

**DOI:** 10.3390/ani16040641

**Published:** 2026-02-17

**Authors:** Jingyue Huang, Haitao Ma, Xixi Duan, Zonglu Wei, Yinjie Zhang, Tao Zhang, Xiaoqun Chen, Yanping Qin, Jun Li, Ziniu Yu, Ying Pan, Yuehuan Zhang

**Affiliations:** 1 Key Laboratory of Aquatic Healthy Breeding and Nutrition Regulation of Guangxi Universities, College of Animal Science and Technology, Guangxi University, Nanning 530004, China; 18897725023@163.com (J.H.); 18239128378@163.com (X.D.); weizonglu@yeah.net (Z.W.); m17865660188@163.com (Y.Z.); 2 Key Laboratory of Breeding Biotechnology and Sustainable Aquaculture (CAS), Key Laboratory of Tropical Marine Bio-Resources and Ecology, Guangdong Provincial Key Laboratory of Applied Marine Biology, South China Sea Institute of Oceanology, Chinese Academy of Sciences, Guangzhou 510301, China; htma@scsio.ac.cn (H.M.); zzhangtao98@163.com (T.Z.); cxq5320@stu.ouc.edu.cn (X.C.); qinyanping@scsio.ac.cn (Y.Q.); jun.li@scsio.ac.cn (J.L.); carlzyu@scsio.ac.cn (Z.Y.); 3 Daya Bay Marine Biology Research Station, Chinese Academy of Sciences, Shenzhen 518124, China; 4 Shenzhen Institute of Guangdong Ocean University, Shenzhen 518120, China; 5 Agro-Tech Extension Center of Guangdong Province, Guangzhou 510145, China; 6 Hainan Provincial Key Laboratory of Tropical Marine Biology Technology, Marine Eco-Environment Engineering Research Institute, Tropical Marine Biological Research Station in Hainan, Chinese Academy of Sciences, Sanya 572024, China

**Keywords:** species identification, COI, *Onchidium stuxbergi*, *Peronia verruculata*, *Onchidium reevesii*, *Platevindex martensi*, *Paromoionchis tumidus*

## Abstract

To address the need for accurate onchidiid slug species identification, the present study engineered a multiplex PCR-based assay to facilitate the efficient, economical, and discrimination of five morphologically similar onchidiid slug species endemic to the South China Sea: *Onchidium stuxbergi*, *Peronia verruculata*, *Onchidium reevesii*, *Platevindex martensi* and *Paromoionchis tumidus*. A diagnostic multiplex PCR assay was designed based on a partial fragment of the mitochondrial cytochrome oxidase subunit I. It utilizes a shared forward primer and five taxon-specific reverse primers to simultaneously produce amplicons of varying sizes, each indicative of a particular species. Five species of Onchidiidae can be identified in a single reaction, which provides technical support for molecular identification of Onchidiidae. The development of a simple, effective and rapid multiple PCR identification method is of great significance for stock management and germplasm conservation of Onchidiidae.

## Simple Summary

The species of Onchidiidae boast a global distribution, with a particularly high concentration of diversity in Southeast Asia. In this review, based on the mitochondrial COI gene sequence, a multiplex PCR method was successfully established for the first time to simultaneously identify five species of Onchidiidae. By designing specific primer combinations, this method can produce species-specific and different-length amplified fragments in a single reaction, and achieve accurate and stable identification of *O. stuxbergi*, *P. verruculata*, *O. reevesii*, *P. martensi* and *P. tumidus*, and no intraspecific variation was observed. The establishment of this technology effectively overcomes the application limitations of traditional morphological identification in larvae or characteristic missing samples, as well as the shortcomings of DNA sequencing technology, such as high cost, long time-consuming and strong equipment dependence. With its strong specificity, simple operation, rapid and economical characteristics, this method provides an efficient and reliable technical tool for large-scale screening, on-site rapid identification and resource monitoring of snail species, and has important practical significance for germplasm resource management, artificial breeding and ecological protection of Onchidiidae.

## 1. Introduction

Multiplex PCR is a technique designed to co-amplify several distinct DNA targets within one reaction mixture using multiple specific primer sets. A PCR technique for amplifying multiple target fragments with primers for multiple DNA templates or different regions of the same template [1]. High interspecies SNP frequency enables precise genotyping through methods like primer extension or PCR assays designed for specific alleles. Multiplex PCR is a simple SNP genotyping strategy. When multiplex PCR is applied to allele-specific or species-specific SNP, its specific amplification products can be directly detected by conventional agarose gel electrophoresis, which enables a relatively low-cost and efficient species identification compared to other molecular methods such as DNA sequencing. At present, this method has been widely used in marine organism species identification research [2,3,4,5].

Onchidiidae belongs to Mollusca, Gastropoda, Systellommatophora. The species of Onchidiidae boast a global distribution, with Southeast Asia exhibiting the highest diversity [6]. The coastal regions of South China, in particular, serve as a prime example of this botanical abundance, being primarily inhabited by *Onchidium stuxbergi* (Westerlund, 1883) [7], *Peronia verruculata* (Cuvier, 1830) [8], *Onchidium reevesii* (Gray, 1850) [9], *Platevindex martensi* (Plate, 1893) [10] and *Paromoionchi tumidus* (Semper, 1880) [11]. Most onchidiid slugs inhabit in the coastal intertidal zone (notable mangroves), with their activity restricted to low tides by tidal rhythms [12]. Onchidiid slug has a high meat yield. Its muscle tissue is rich in protein, and contains a high level of essential amino acids and a variety of mineral components, indicating that this species is a marine biological resource with excellent nutritional quality and health promotion potential. Notably, comparative studies have demonstrated significant interspecific differences in lipid composition among onchidiids [13]. For instance, key species such as *P. verruculata* and *O. reevesii* are particularly rich in essential fatty acids, while others exhibit distinct profiles of polyunsaturated fatty acids. These findings highlight that the nutritional and economic value is highly species-dependent. In addition, it has potential medicinal value in the treatment of liver cirrhosis, relieving asthma, improving digestive function and protecting eyesight, and has important development and application prospects [14]. Therefore, accurate species identification is not only a taxonomic necessity but also a prerequisite for the precise assessment, sustainable management, and optimal utilization of these valuable biological resources.

As a representative group of the evolution of ocean to land radiation, Onchidiidae is an ideal model for analyzing the molecular mechanism of terrestrial adaptation [15,16]. At the same time, it is an aquaculture species with important economic and ecological value [17]. However, accurate species identification is fundamental to utilizing the species for other researchs. At present, the molecular identification of onchidiid slugs mainly depends on DNA sequencing technology. For example, Dayrat et al. [18] found a new species, *Onchidium melakense* (Dayrat and Goulding, 2019) [19], by analyzing mitochondrial and nuclear DNA sequences. Goulding et al. [15] reconstructed the phylogenetic relationship of onchidiid slug based on three mitochondrial markers and three nuclear markers. However, DNA sequencing methods have the limitations of high cost and long cycles, and it is difficult to apply them to the high-throughput screening of large-scale samples. In contrast, multiplex PCR technology has the significant advantages of high efficiency and low cost. It can not only achieve rapid identification of large quantities of samples, but also show high detection sensitivity for trace or degraded DNA templates.

Although Onchidiidae comprises numerous species and is widely distributed, there are still obvious deficiencies in its species cognition [12]. The morphological characteristics of many onchidiid species have not been fully studied. Furthermore, their external morphology is highly plastic, being significantly influenced by environmental conditions and ontogenetic stage, which makes species distinction based solely on such traits notoriously difficult, if not impossible, despite the potential utility of internal anatomical characters [20]. The morphological identification method is not only greatly affected by subjective judgment, but also, particularly for larvae or morphologically cryptic species, the process can be time-consuming and yield low reliability. The multiplex PCR method used in this study, based on the mitochondrial COI gene sequence, can effectively overcome the limitations posed by phenotypic plasticity, achieve identification of closely related species, and is suitable for sample types such as larvae and specimens that are difficult to morphologically identify.

In recent years, the population of Onchidiidae has declined sharply, largely due to the overexploitation of coastal beaches and the intensification of marine pollution [21]. The research on Onchidiidae mainly focuses on ecology [22,23,24], morphology [25], nervous system [26], genetic diversity, and population differentiation [6,12,15,18,20,21]. In this study, a multiplex species-specific PCR method was established for the first time to rapidly and accurately identify five species of *Onchidium* in the coastal area of South China.

## 2. Materials and Methods

### 2.1. Primer Design

Species-specific PCR primers were designed using mitochondrial COI gene. The components of the multiplex PCR reaction system are shown in Table 1. We downloaded the sequences of all known onchidiid slug species from GenBank, combined with the sequences we obtained from more than 150 onchidiid slugs collected from China (Table 2). The analysis incorporated the full spectrum of genetic variants, integrating every accessible haplotype for a given species into the study. The COI sequences included *O. stuxbergi* (MZ831962, Bravo, 2021), *P. verruculata* (NC068813, Wang, 2023), *O. reevesii* (OP714175, Wang, 2023), *P. martensi* (MZ832009, Bravo, 2021) and *P. tumiclus* (MH054945, Dayrat, 2019). 

Sequences from all five target species were aligned using BioEdit v7.2.5 (Hall, Carlsbad, CA, USA) with its built-in ClustalW v2.1 algorithm (Thompson, Dublin, Ireland) under default parameters. The resulting alignment was manually curated to identify fixed, species-specific single-nucleotide polymorphisms (SNPs) within conserved regions. These diagnostic SNPs were strategically selected to serve as the 3′-end sequences for the species-specific reverse primers, thereby conferring amplification specificity. To facilitate amplification across multiple taxa, a universal forward primer (F) was engineered for concurrent application in all five target species. Correspondingly, based on the alignment, species-specific reverse primers (R-ost, R-pve, R-ore, R-pma, R-ptu) were designed, with the determinant SNP incorporated at the 3′ terminus of each primer (Table 1). Prior to experimental validation, in silico specificity of each primer was assessed via BLASTN against the NCBI nucleotide database. Each species-specific reverse primer showed perfect identity (100%) to its intended target, effectively supporting its theoretical specificity and ruling out potential cross-reactivity in silico.

### 2.2. Species Collection

**Figure 1:**
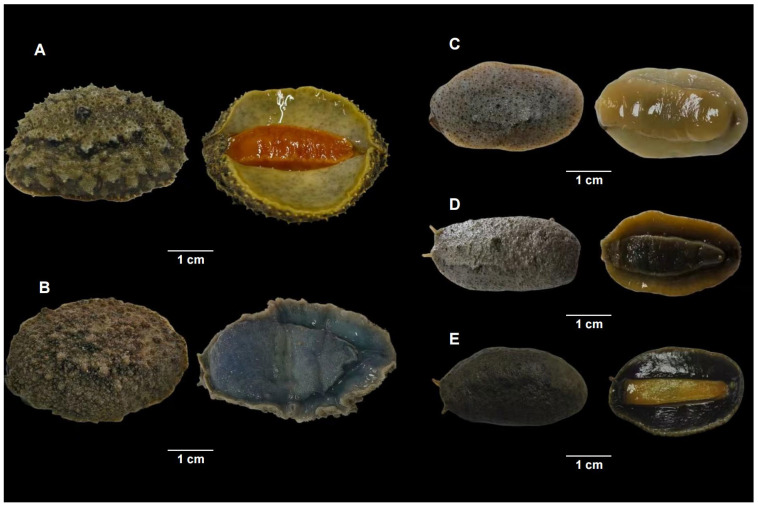
External morphology of the five species of *Onchidium*. (**A**) *Onchidium stuxbergi*, (**B**) *Peronia verruculata*, (**C**) *Onchidium reevesii*, (**D**) *Paromoionchi tumidus*, (**E**) *Platevindex martensi*.

**Figure 2:**
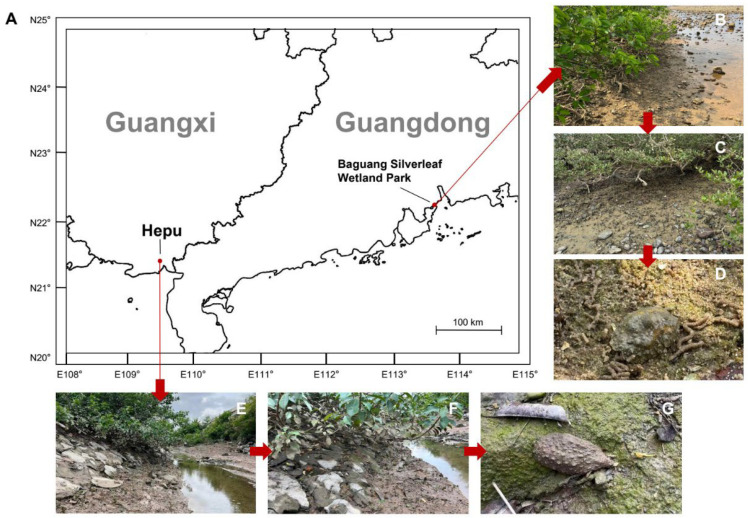
The sampling positions and habitats of onchidiid slug. (**A**) Two sampling positions of onchidiid slug; (**B**–**D**) Baguang Silverleaf Wetland Park, Shenzhen, Guangdong, China (22°12′ N, 113°36′ E); (**E**–**G**) Zhakou Town, Hepu County, Beihai City, Guangxi, China (21°24′ N, 109°30′ E).

In this study, validation procedures were carried out using specimens from five distinct species of onchidiid slugs (Figure 1). Thirty samples were tested for each species (Table 2). Where feasible, individuals were sampled from multiple populations per species. All specimens originated from the local market in Guangdong and Guangxi Provinces, China (Figure 2). Species identification for the five onchidiid slugs was based on muscle tissue and external morphological characteristics [20].

### 2.3. DNA Extraction and PCR Amplification

DNA was isolated from adductor muscle tissue that had been preserved in ethanol using the Magen HiPure Mollusc DNA kit (Magen, Guangzhou, China). In the multiplex PCR, the universal forward primer was used at a final concentration of 0.2 μM. The species-specific reverse primers were used at 0.05 μM (R-ost, R-pve, R-pma, R-ptu) and 0.1 μM (R-ore). PCR reactions were conducted in a total volume of 25 microliters utilizing an ABI Veriti 96-well thermal cycler. Reaction parameters were refined through systematic evaluation of various primer combinations and annealing temperatures. The finalized reaction mixture formulation is documented in Table 1. The thermal cycling protocol consisted of: initial denaturation at 94 °C for 180 s, followed by denaturation at 94 °C for 30 s, annealing at 60 °C for 30 s, and extension at 72 °C for 60 s, concluding with extension at 72 °C for 180 s and preservation at 4 °C. Each PCR process contained a negative control (no template). Amplified DNA fragments were electrophoresed through a 2.75% agarose impregnated with 1.0 μg/mL ethidium bromide. The resulting bands were visualized under UV illumination, and species differentiation was achieved by analyzing the varying amplicon sizes.

## 3. Results

### 3.1. Identification of Five Species of Onchidiid Slug Using COI

**Figure 3:**
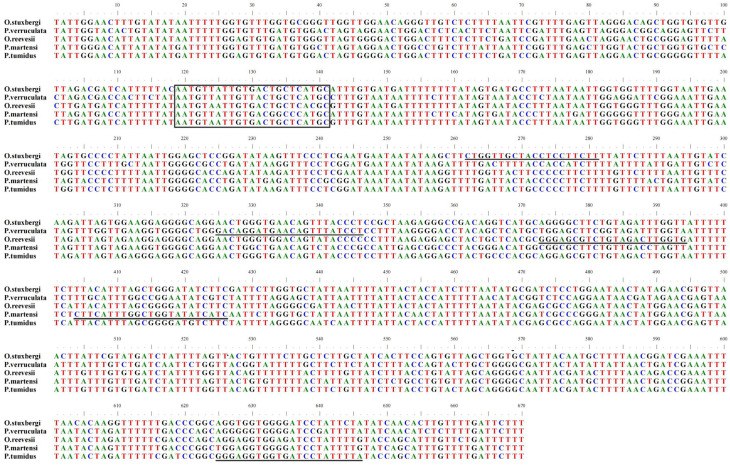
COI gene sequence alignment of five Onchidiidae species: *O. stuxbergi*, *P. verruculata*, *O. reevesii*, *P. martensi* and *P. tumidus*. The box indicates a common forward primer sequence. Species-specific primer sites are marked with underlines.

The COI gene exhibits substantial sequence divergence across onchidiid taxa. For primer construction, we targeted conserved regions that demonstrated fixed interspecific nucleotide differences while displaying complete intraspecific conservation. Primer R-ost, designed for *O. stuxbergi* specificity, incorporated eight diagnostic sites (262, 265, 266, 268, 269, 271, 274, 277). Primer R-pve, designed for *P. verruculata* specificity, incorporated seven diagnostic sites (325, 328, 331, 334, 340, 343, 346). Primer R-ore, designed for *O. reevesii* specificity, incorporated nine diagnostic sites (373, 376, 379, 382, 385, 388, 389, 391, 394). Primer R-pma, designed for *P. martensi* specificity, incorporated seven diagnostic sites (404, 406, 412, 415, 418, 421, 424). Primer R-ptu, designed for *P. tumidus* specificity, incorporated seven diagnostic sites (625, 628, 634, 640, 644, 646, 649) (Figure 3).

**Figure 4:**
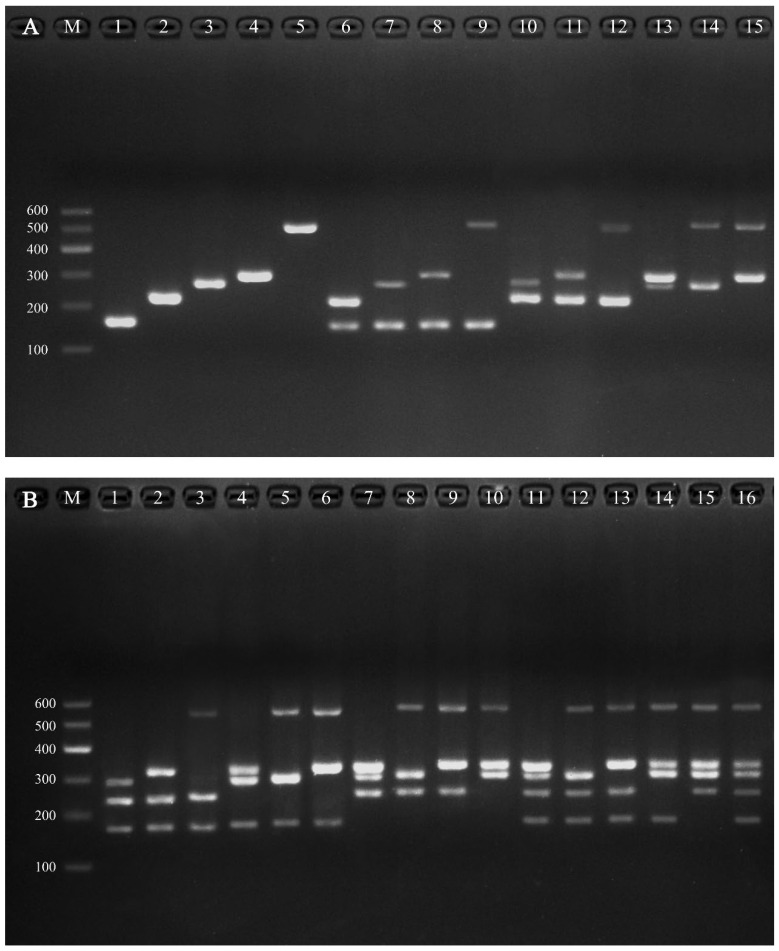
Multiplex species-specific PCR with 5 primers from the COI gene. Note: (**A**): 1. *O.stuxbergi* (162 bp); 2. *P. verruculata* (227 bp); 3. *O. reevesii* (275 bp); 4. *P. martensi* (307 bp); 5. *P. tumidus* (527 bp); 6. *O.stuxbergi* + *P. verruculata*; 7. *O. stuxbergi* + *O. reevesii*; 8. *O. stuxbergi* + *P. martensi*; 9. *O. stuxbergi* + *P. tumidus*; 10. *P. verruculata* + *O. reevesii*; 11. *P. verruculata* + *P. martensi*; 12. *P. verruculata* + *P. tumidus*; 13. *O. reevesii* + *P. martensi*; 14. *O. reevesii* + *P. tumidus*; 15. *P. martensi* + *P. tumidus*; M. 600 bp DNA ladder. (**B**): 1. *O. stuxbergi* + *P. verruculata* + *O. reevesii*; 2. *O. stuxbergi* + *P. verruculata* + *P. martensi*; 3. *O. stuxbergi* + *P. verruculata* + *P. tumidus*; 4. *O. stuxbergi* + *O. reevesii* + *P. martensi*; 5. *O. stuxbergi* + *O. reevesii* + *P. tumidus*; 6. *O. stuxbergi* + *P. martensi* + *P. tumidus*; 7. *P. verruculata* + *O. reevesii* + *P. martensi*; 8. *P. verruculata* + *O. reevesii* + *P. tumidus*; 9. *P. verruculata* + *P. martensi* + *P. tumidus*; 10. *O. reevesii* + *P. martensi* + *P. tumidus*; 11. *O. stuxbergi* + *P. verruculata* + *O. reevesii* + *P. martensi*; 12. *O. stuxbergi* + *P. verruculata* + *O. reevesii* + *P. tumidus*; 13. *O. stuxbergi* + *P. verruculata* + *P. martensi* + *P. tumidus*; 14. *O. stuxbergi* + *O. reevesii* + *P. martensi* + *P. tumidus*; 15. *P. verruculata* + *O. reevesii* + *P. martensi* + *P. tumidus*; 16. *O. stuxbergi* + *P. verruculata* + *O. reevesii* + *P. martensi* + *P. tumidus*; M. 600 bp DNA ladder.

After optimization, a COI-based multiplex PCR employing six primers (one common forward and five unique reverse primers) reliably amplified DNA from all five species. The results showed that the designed reverse primers exhibited specific amplification solely in their corresponding target species, and the size of the obtained fragments was consistent with expectations: approximately 162 bp in *O. stuxbergi*, 227 bp in *P. verruculata*, 275 bp in *O. reevesii*, 307 bp in *P. martensi*, and 527 bp in *P. tumidus* (Figure 4; Table 3). All five target species could be accurately identified by a single PCR. The difference between the 3′ end sequence of the reverse primer and the COI sequence of the non-target species avoids interspecific cross-reaction and intraspecific variation.

### 3.2. Intraspecific Variation

To evaluate the consistency of the assays and investigate potential genetic diversity within individual species, non-destructive sampling detection was performed on 30 individuals of each of the five target species. All onchidiid slugs were positive, and the amplified fragment size remained highly consistent within the species, confirming that the method had good repeatability and stability. Figures are not provided due to their high similarity to Figure 4.

## 4. Discussion

In this study, primers were designed within the partial sequence of mitochondrial COI gene, and a multiplex PCR method for the identification of five species of Onchidiidae was established. The primer combination amplified a species-specific fragment in the COI gene region: 162 bp for *O. stuxbergi*, 227 bp for *P. verruculata*, 275 bp for *O. reevesii*, 307 bp for *P. martensi*, 527 bp for *P. tumidus*. All five species were clearly identified in a single multiplex PCR reaction. All individuals of each species produced the expected size of the amplified product, and no intraspecific variation was observed. Each sample was accurately assigned to one of the five target species.

The two core elements of creating a multiplex PCR assay are to identify diagnostic sequence regions and to select species-specific single-nucleotide polymorphisms (SNPs) within these regions for primer design. The COI gene is widely used for species discrimination due to its substantial interspecific sequence variation. This feature is essential for screening multiple diagnostic for primer design. The COI gene fragment has a high mutation rate, which is suitable for the study of species classification and genetic relationship among related species [27,28]. A study also found that COI can obtain a high horizontal resolution of onchidiid slugs [20]. Therefore, we can diagnose a large number of diagnostic SNPs even between genetically similar species like *O. stuxbergi* and *O. reevesii* by selecting COI.

Although DNA sequencing is the most reliable and accurate method for species identification, its high cost and time consumption limit its application in large-scale routine detection [3]. Multiplex PCR achieves high-throughput parallel detection by using multiple pairs of specific primers to achieve co-amplification of several gene loci in one reaction system, which significantly reduces reagent consumption and sample demand [29]. This method only requires a conventional PCR instrument and an agarose gel electrophoresis apparatus. Thereby, it eliminates the need for the expensive sequencing services and the subsequent sequence alignment and phylogenetic analysis typically required for definitive identification via DNA sequencing [30]. Therefore, in large-scale sample screening, on-site rapid identification and species identification under resource-limited conditions, multiplex PCR has become a more advantageous technical choice due to its strong directionality, rapid detection and economic efficiency.

We established a multiplex PCR system for the first time and successfully identified five onchidiid slug species along the coast of South China. The development of this multiplex PCR system has achieved efficient and convenient species identification, especially for the samples with missing morphological characteristics or in the juvenile stage. Its low cost and rapid detection characteristics can support regulators to implement species monitoring, which is of great significance for the development of breeding technology and resource protection of onchidiid slugs.

## 5. Conclusions

In this study, a multiplex PCR-based detection method was designed for the first time to facilitate the efficient, economical, and discrimination of five morphologically similar onchidiid slug species endemic to the South China Sea: *Onchidium stuxbergi*, *Peronia verruculata*, *Onchidium reevesii*, *Platevindex martensi* and *Paromoionchis tumidus*. A diagnostic multiplex PCR assay was designed around the mitochondrial COI locus. The development of a simple, effective and rapid multiple PCR identification method is of great significance for stock management and germplasm conservation of Onchidiidae.

## Figures and Tables

**Table 1:** The PCR components of multiplex PCR assay.

PCR Components	Content (μL)
dNTP Mixture	4
10 × buffer	2.5
MgCl_2_	1
Forward primer	2
Reverse primer (contains the following components)	
*Onchidium stuxbergi*	0.5
*Peronia verruculata*	0.5
*Onchidium reevesii*	1
*Platevindex martensi*	0.5
*Paromoionchi tumidus*	0.5
Template DNA	3.8
Taq polymerase	0.5
Water	8.2

**Table 2:** Onchidiid slug species, collection site, size and number for the validation of multiplex species-specific PCR.

Species	Collection Site	Size	Number
*Onchidium stuxbergi*	Beihai, Guangxi province, China	Body length 4–6 cm	30
*Peronia verruculata*	Beihai, Guangxi province, China	Body length 4–6 cm	30
*Platevindex martensi*	Beihai, Guangxi province, China	Body length 4–5 cm	30
*Onchidium reevesii*	Shenzhen, Guangdong province, China	Body length 4–5 cm	30
*Paromoionchi tumidus*	Beihai, Guangxi province, China	Body length 3–4 cm	30

**Table 3:** Summary of five species-specific primers for species distinction in *Onchidiidae*.

Primer	Specificity	Primer Sequence (5′-3′)	Size (bp)
COforward	All	AATGTTATTGTGACTGCTCATGC	
COOst162r	*Onchidium.stuxbergi*	AAGAAGGAGGTAGCAACCAG	162
COPve227r	*Peronia.verruculata*	AGGATAAACTGTTCATCCTGTC	227
COOre275r	*Onchidium.reevesii*	CACCAAGTCTACAGACGCTCCC	275
COPma307r	*Platevindex.martensi*	GATGATATACCAGCCAAATGAAG	307
COPtu527r	*Paromoionchi.tumidus*	TAAAATAGGATCACCACCTCCC	527

## Data Availability

Data will be made available on request.

## References

[1] Chamberlain J.S., Gibbs R.A., Rainer J.E., Nguyen P.N., Thomas C. (1988). Deletion screening of the Duchenne muscular dystrophy locus via multiplex DNA amplification. Nucleic Acids Res..

[2] Larsen J.B., Frischer M.E., Rasmussen L.J., Hansen B.W. (2005). Single-step nested multiplex PCR to differentiate between various bivalve larvae. Mar. Biol..

[3] Wang H., Guo X. (2008). Identification of *Crassostrea ariakensis* and Related Oysters by Multiplex Species-Specific PCR. J. Shellfish Res..

[4] Melo M.A.D., Da Silva A.R.B., Beasley C.R., Tagliaro C.H. (2013). Multiplex species-specific PCR identification of native and non-native oysters (*Crassostrea*) in Brazil: A useful tool for application in oyster culture and stock management. Aquac. Int..

[5] Ma H., Gao H., Zhang Y., Qin Y., Xiang Z., Li J., Zhang Y., Yu Z. (2021). Multiplex species-specific PCR identification of native giant clams in the South China Sea: A useful tool for application in giant clam stock management and forensic identification. Aquaculture.

[6] Dayrat B., Goulding T.C., Khalil M., Apte D., Tan S.H. (2019). A new species and new records of *Onchidium* slugs (*Gastropoda*, *Euthyneura*, *Pulmonata*, *Onchidiidae*) in South-East Asia. ZooKeys.

[7] Westerlund C.A. (1883). Malakologische Miscellen II. Nachrichtsblatt Dtsch. Malakozool. Ges..

[8] Cuvier G. (1830). Le Règne Animal Distribué D’après son Organisation.

[9] Gray J.E. (1850). Figures of Molluscous Animals Selected from Various Authors. Etched for the Use of Students.

[10] Plate L. (1893). Studien über opisthopneumone Lungeschnecken, II, Die Oncidiidien. Zool. Jahrb..

[11] Semper C., Kreidel C.W. (1885). Dritte Familie, Onchidiidae. Reisen Im Archipel Der Philippinen, Wissenschaftliche Resultate.

[12] Goulding T.C., Khalil M., Tan S.H., Dayrat B. (2018). Integrative taxonomy of a new and highly-diverse genus of onchidiid slugs from the Coral Triangle (*Gastropoda*, *Pulmonata*, *Onchidiidae*). ZooKeys.

[13] Yao L.X., Yang T.Z., Zhu M., Shen H.D., Li B.H., Diao Y. (2016). Lipid and Fatty Acid Composition Analysis of Four Species of *Onchidiidae*. J. Fish. China.

[14] Wang B., Chen D., Yu M., Liu Y., Liu P., Zhang X. (2021). A Review on Metabolites from *Onchidium* Genus: Chemistry and Bioactivity. Chem. Biodivers..

[15] Goulding T.C., Khalil M., Tan S.H., Cumming R.A., Dayrat B. (2022). Global diversification and evolutionary history of onchidiid slugs (*Gastropoda*, *Pulmonata*). Mol. Phylogenet Evol..

[16] Klussmann-Kolb A., Dinapoli A., Kuhn K., Streit B., Albrecht C. (2008). From sea to land and beyond—New insights into the evolution of euthyneuran *Gastropoda* (Mollusca). BMC Evol. Biol..

[17] Ruthensteiner B., Schaefer K. (2002). The cephalic sensory organ in veliger larvae of pulmonates (*Gastropoda*: Mollusca). J. Morphol..

[18] Dayrat B., Goulding T.C., Khalil M., Apte D., Bourke A., Comendador J., Tan S.H. (2019). A new genus and three new species of mangrove slugs from the Indo-West Pacific (Mollusca: *Gastropoda*: *Euthyneura*: *Onchidiidae*). Eur. J. Taxon..

[19] Dayrat B., Goulding T.C. (2019). Systematics of the onchidiid slug *Onchidina australis* (Mollusca: *Gastropoda*: *Pulmonata*). Arch. Molluskenkd..

[20] Sun B., Chen C., Shen H., Zhang K., Zhou N., Qian J. (2014). Species diversity of *Onchidiidae* (Eupulmonata: Heterobranchia) on the mainland of China based on molecular data. Molluscan Res..

[21] Zhou N., Shen H., Chen C., Sun B., Zheng P., Wang C. (2016). Genetic structure of *Onchidium* “*struma*” (Mollusca: Gastropoda: Eupulmonata) from the coastal area of China based on mt *CO* I. Mitochondrial DNA A.

[22] Li X., Jia L., Zhao Y., Wang Q., Cheng Y. (2009). Seasonal bioconcentration of heavy metals in *Onchidium struma* (Gastropoda: Pulmonata) from Chongming Island, the Yangtze Estuary, China. J. Environ. Sci..

[23] Shen H., Li K., Chen H., Chen X., He Y., Shi Z. (2011). Experimental ecology and hibernation of *Onchidium struma* (Gastropoda: Pulmonata: Systellommatophora). J. Exp. Mar. Biol. Ecol..

[24] Zhou Z., Wu Q., Xie Q., Ling C., Zhang H., Sun C., Ju J. (2020). New Borrelidins from *Onchidium* sp. Associated *Streptomyces olivaceus* SCSIO LO13. Chem. Biodivers..

[25] Xu G., Yang T., Wang D., Li J., Liu X., Wu X., Shen H. (2018). A comprehensive comparison of four species of *Onchidiidae* provides insights on the morphological and molecular adaptations of invertebrates from shallow seas to wetlands. PLoS ONE.

[26] Shimotsu K., Nishi T., Nakagawa S., Gotow T. (2010). A new role for photoresponsive neurons called simple photoreceptors in the sea slug *Onchidium verruculatum*: Potentiation of synaptic transmission and motor response. Comp. Biochem. Phys. A.

[27] Hebert P.D.N., Cywinska A., Ball S.L., Waard J.R. (2003). Biological identifications through DNA barcodes. Proc. R. Soc. Lond. Ser. B.

[28] Nuryanto A., Duryadi D., Soedharma D., Blohm D. (2007). Molecular Phylogeny of Giant Clams Based on Mitochondrial DNA Cytochrome C Oxidase I Gene. HAYATI J. Biosci..

[29] Markoulatos P., Siafakas N., Moncany M. (2002). Multiplex polymerase chain reaction: A practical approach. J. Clin. Lab. Anal..

[30] Shendure J., Balasubramanian S., Church G.M., Gilbert W., Rogers J., Schloss J., Waterston R. (2017). DNA sequencing at 40: Past, present and future. Nature.

